# Oral lichen planus and HPV lesions

**DOI:** 10.11604/pamj.2018.29.74.14546

**Published:** 2018-01-24

**Authors:** Cinzia Casu, Luca Viganò

**Affiliations:** 1 Private Dental Practice, Cagliari, Italy; 2 University of Milan, Department of Radiology, Milano, Italy

**Keywords:** Oral lichen planus, oral HPV lesions, oral leucoplakia

## Image in medicine

A 42 years old male patient went to our observation for a white lesion in the left cheek presented 5 months ago. He reported general good health, a previous diagnosis of genital lichen planus. During the clinical examination we observed a white not scrapable lesion of 3 cm in the left cheek and 2 small esophitic lesions in the gingival tissue near the retromolar trigone, with cauliflower shape. The lesions is not linked with a traumatic event and we decided to make an incisional biopsy and 2 excisional biopsies for esophitic lesions on the gingiva, with a temporary diagnosis of leukoplakia and HPV squamous papilloma. The histological examination confirms instead the presence of an oral plaque lichen planus, without signs of dysplasia and the presence of 2 squamous papillomas on the tissue removed in the gingival. It is the first documented case in literature of an oral lichen planus which occurs many years after genital lesions, rare in a male subject. In addition, the simultaneous presence of HPV lesions in the same oral anatomic region is particular. Treatment includes topical therapy with podophyllin for HPV lesions or surgical excision and medical treatment of lichen planus with topical cortisones, immunosuppressants or aloe vera-based gels.

**Figure 1:**
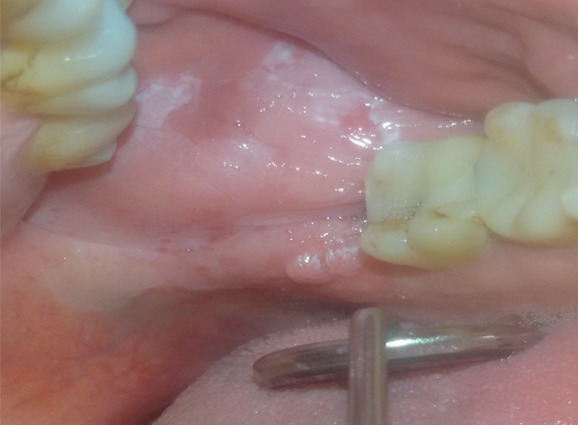
oral lichen planus and in the cheek and HPV gengival lesions

